# Association between WeChat-based remote care guidance and diabetic foot ulcer healing: a retrospective cohort study

**DOI:** 10.7717/peerj.20624

**Published:** 2026-01-09

**Authors:** De Qin Chen, Chao Yun Jiang, Tian Hong Cai, Rong Zhang, Teng Hui Zhan

**Affiliations:** 1 Vascular Surgery & Interventional Medicine, Fujian Maternity and Child Health Hospital, Fuzhou, Fujian, China; 2 College of Clinical Medicine for Obstetrics & Gynecology and Pediatrics, Fujian Medical University, Fuzhou, Fujian, China

**Keywords:** Diabetic foot ulcer, Remote care, Mobile health, Wound healing

## Abstract

**Objective:**

The aim of this study was to evaluate the effectiveness of WeChat-based remote care guidance as a supplement to standard care for patients with diabetic foot ulcers. Our specific objectives were to compare healing rates, self-management behaviors, and patient satisfaction between the two groups, with a focus on identifying patient subgroups that might benefit most from this approach.

**Methods:**

A retrospective cohort study was conducted at Fujian Maternity and Child Health Hospital between June 2021 and December 2022, with follow-up until December 2024. Among 131 eligible patients with diabetic foot ulcers (Wagner grades 1–4), 59 received WeChat -based guidance (intervention) while 72 received standard care (control). Primary outcomes included wound healing rate and time-to-healing. Quality of life (WHOQOL-BREF) and treatment satisfaction (DTSQs) were assessed at baseline, 3, 6, 12, and 24 months. Analyses were adjusted for demographic characteristics, clinical parameters, and disease severity indicators.

**Results:**

The intervention group showed significantly higher healing rates (88.1% *vs* 63.9%, *P* = 0.001) and faster healing time (HR = 2.27, 95% CI [1.35–3.82], *P* = 0.002). The effect was particularly pronounced in Wagner grade 2–3 ulcers (HR = 14.3–34.2, *P* < 0.001) and patients receiving interventional procedures (HR = 3.4, 95% CI [1.8–6.3], *P* <0.001). At 24 months, the intervention group demonstrated greater improvements in quality of life (mean difference = 7.87, *P* < 0.001) and treatment satisfaction (mean difference = 6.70, *P* < 0.001).

**Conclusion:**

WeChat-based remote care guidance was associated with better diabetic foot ulcer healing outcomes, particularly for moderate-severity ulcers and patients undergoing interventional procedures. Our findings also suggest associations between this approach and improvements in quality of life and treatment satisfaction.

## Introduction

Diabetic foot ulcers (DFUs) are one of the most serious complications of diabetes, severely affecting patients’ quality of life and increasing the burden of care. According to the most recent systematic review and meta-analysis, the overall prevalence of diabetic foot ulcers worldwide is 6.3% (95% CI [5.4–7.3%]), with men (4.5%) having a higher prevalence than women (3.5%) (Zhang et al., 2017; Zaidi et al., 2024). In terms of wound healing rates, with standard treatment, approximately 60–70% of diabetic foot ulcers can heal within 12 months, but 15–20% of patients may still require amputation treatment (Oliveira-Cortez et al., 2023; Ghanassia et al., 2008). DFUs significantly decrease patients’ health-related quality of life compared to non-diabetic patients and diabetic patients without DFUs (Armstrong, Boulton & Bus, 2017).

Remote care guidance *via* the WeChat platform is an innovative model of doctor-patient interaction based on mobile medical technology. It provides continuous medical guidance and management for patients with diabetic foot ulcers through real-time image transmission, online consultation and health education. Studies have shown that this model can significantly improve patient compliance and self-management skills (Yang et al., 2021). In the management of chronic diseases, WeChat remote management has shown significant results, such as improving medication adherence in chronic rhinosinusitis patients after functional endoscopic sinus surgery, enhancing disease control and therapy outcomes in cough-variant asthma patients, and increasing follow-up satisfaction in patients with head and neck tumors (Chen et al., 2020).

Currently, there is still controversy regarding the impact of WeChat platform remote management on diabetic foot wound healing. Some studies have shown that remote management can shorten healing time and reduce the risk of complications; however, other studies have pointed out that the effect of remote management may vary from person to person due to factors such as patient age, education level, and ability to use smart devices. In addition, most of the existing studies are short-term observational and there is a lack of long-term follow-up data to support them.

For this reason, we propose to conduct this retrospective cohort study to evaluate the effect of WeChat platform remote management on wound healing in patients with diabetic foot ulcers receiving care at our institution, including both inpatient and outpatient services. This study has the following characteristics: it is the first time in the region to conduct long-term follow-up observation for 2 years; it adopts a standardised remote management process and wound assessment method. The results of the study will provide an important basis for formulating a remote management strategy for diabetic foot and may provide new ideas for improving patient outcomes.

## Materials and Methods

### Study design and population

We conducted a retrospective cohort study at Fujian Maternity and Child Health Hospital between June 2021 and December 2022, with follow-up until December 2024. Patients receiving treatment for diabetic foot were identified through electronic medical records. Of the 131 patients who met eligibility criteria, 59 utilized the WeChat-based management platform (intervention group) while 72 received standard care without WeChat support (control group). This retrospective cohort study examined outcomes in patients with diabetic foot ulcers who received either standard care alone or standard care supplemented with WeChat-based remote monitoring. The exposure factor was defined as participation in the WeChat-based remote care program, and the primary outcome was ulcer healing rate at 16 weeks. Patients were not randomly assigned to groups. Rather, during the study period (January 2021–December 2022), our center offered WeChat-based remote care as an optional supplement to standard care. Patients who opted to participate in this program constituted the exposure group, while those who received standard care alone formed the non-exposure group. This assignment reflects real-world clinical practice rather than experimental allocation. All patients in both groups received identical standard care according to international guidelines for diabetic foot management, including wound debridement, infection control with appropriate antibiotics when indicated, offloading techniques, vascular assessment and intervention when necessary, blood glucose management, and regular dressing changes. Based on previous studies in our center showing a standard care healing rate of approximately 40% at 16 weeks, we calculated that a minimum of 58 patients per group would provide 80% power to detect a clinically meaningful 25% increase in healing rate (from 40% to 65%) with a two-sided alpha of 0.05. The final sample of 131 patients (59 in the WeChat group and 72 in the standard care group) was therefore adequately powered for our primary analysis.

Inclusion criteria were: (1) age ≥18 years; (2) clinically diagnosed diabetic foot ulcers (Wagner grades 1–4); (3) clinically stable condition not requiring immediate hospitalization; and (4) willingness to participate and provide informed consent. For the intervention group, additional criterion included proficiency in WeChat use by patient or primary caregiver.

Exclusion criteria included: (1) Wagner grade 5 ulcers; (2) severe cardiac, hepatic, or renal dysfunction; (3) severe psychiatric disorders preventing compliance; (4) terminal malignancy; (5) life expectancy shorter than study period; (6) death during follow-up period; and (7) severe communication barriers affecting remote interaction.

### Remote nursing guidance based on WeChat platform

#### Training of team members

The multidisciplinary intervention team comprised one board-certified vascular surgeon and three certified vascular nursing specialists with extensive experience in diabetic foot care. Prior to implementation, all team members underwent a comprehensive training programme developed in accordance with international telemedicine guidelines and validated by an expert panel in diabetic foot care. The training curriculum integrated essential competencies in digital healthcare delivery and specialized clinical expertise. Team members received intensive instruction in WeChat platform operations, focusing on remote wound image assessment protocols and data security measures. Clinical training emphasized standardized diabetic foot assessment, wound classification, vascular evaluation, and infection recognition protocols. The programme also incorporated advanced patient education methodologies, including wound care instruction, lifestyle modification guidance, and therapeutic compliance enhancement strategies. Additionally, team members were trained in patient-centered communication, crisis intervention, and psychological support techniques to ensure comprehensive care delivery. Technical competencies encompassed platform troubleshooting, image quality optimization, and remote consultation protocols. To maintain quality standards, all team members underwent rigorous competency assessments before initiating remote care services. The intervention protocol mandated regular performance evaluations and continuing education sessions to ensure sustained service quality and updated clinical knowledge in diabetic foot management.

The WeChat-based remote care intervention comprised two integrated components:

Educational Component: The educational component delivered comprehensive diabetic foot management materials including: (1) wound care protocols with visual guides, (2) glycemic control strategies, (3) infection prevention guidelines, (4) therapeutic compliance recommendations, and (5) lifestyle modifications for wound healing. These resources remained continuously accessible to patients through text, video, and interactive formats.

Interactive consultation component: The interactive component provided real-time professional support through a dedicated healthcare team available daily from 8:00 to 20:00. This service facilitated wound care guidance through standardized photography protocols, treatment adherence monitoring, and timely intervention for complications. Healthcare professionals assessed wound images, adjusted treatment plans, and provided personalized feedback.

Additional platform capabilities: The platform incorporated medication and glucose monitoring reminders, secure wound photograph transmission, and weekly moderated peer support group discussions where patients shared experiences while healthcare providers addressed misconceptions.

Integration with clinical care: During clinical visits, healthcare providers integrated platform-based education with traditional care, reviewing online activities, reinforcing key concepts, and assessing patients’ understanding of diabetic foot management protocols.

#### Research tools

WeChat (Weixin in Chinese) is China’s most widely used mobile application with over 1.2 billion monthly active users. Developed by Tencent, it combines instant messaging, social media, and mobile payment capabilities. Its ubiquity in China (installed on over 90% of smartphones) makes it an ideal platform for healthcare interventions, as it requires no additional software installation and offers a familiar interface for patients of all ages.

Patient-reported outcomes were assessed using two validated instruments: the Diabetes Treatment Satisfaction Questionnaire status version (DTSQs) and the World Health Organization Quality of Life-BREF (WHOQOL-BREF).

The DTSQs is a widely validated instrument designed to measure satisfaction with diabetes treatment regimens (Saisho, 2018). It consists of eight items, with six items assessing key aspects of treatment satisfaction: current treatment satisfaction, perceived treatment flexibility, convenience, understanding of diabetes, likelihood of recommending treatment to others, and satisfaction to continue current treatment. Each item is scored on a 7-point scale from 0 (very dissatisfied) to 6 (very satisfied). Two additional items separately evaluate perceived frequency of unacceptably high (hyperglycemia) and low (hypoglycemia) blood glucose levels, also scored from 0 (none of the time) to 6 (most of the time). The total treatment satisfaction score is calculated from the sum of the six satisfaction items, with higher scores indicating greater treatment satisfaction.

The World Health Organization Quality of Life-BREF (WHOQOL-BREF) scale was developed on the basis of the WHOQOL-100 scale (Saisho, 2018). The WHOQOL-BREF consists of 26 items which assess quality of life across four domains: physical health, psychological well-being, social relationships, and environmental factors. The instrument employs a five-point rating scale, with items 3, 4, and 26 reverse-scored. Two further items are employed to provide independent assessments of overall quality of life and general health satisfaction. Domain scores are calculated as the mean of their respective items, with higher scores indicating better quality of life in each domain.

#### Data collection

We collected comprehensive demographic, clinical, and wound-related data from all participants. Demographic information including age, gender, education level, and duration of diabetes was obtained from electronic medical records. Clinical parameters including HbA1c, blood pressure, lipid profiles, renal function tests, and complete blood counts were assessed at baseline and at the 16-week follow-up visit.

Diabetic foot ulcer assessment was performed by trained wound care specialists during scheduled clinic visits at baseline, 3, 6, 12, and 24 months. Complete healing was defined as full epithelialization without drainage maintained for at least 2 weeks. Ulcer size was measured using digital planimetry with sterile transparent film tracings. Wound characteristics including location, depth, presence of infection (according to IDSA criteria), and vascular status (based on ankle-brachial index and duplex ultrasound when indicated) were systematically documented.

Diabetic foot self-care behaviors were evaluated using the validated Diabetic Foot Self-Care Behavior Scale (DFSBS) at baseline, 3, 6, 12, and 24 months. This 15-item questionnaire assesses foot inspection, footwear practices, foot hygiene, and help-seeking behaviors, with higher scores indicating better self-care practices. Patient knowledge regarding diabetic foot care was assessed using a structured questionnaire covering key educational aspects of foot protection and early warning signs.

For patients in the WeChat group, additional monitoring parameters included frequency of remote consultations, image uploads, response times from healthcare providers, and compliance with recommended care activities. Patient engagement metrics were documented throughout the follow-up period through the WeChat platform’s backend database, including frequency of educational content access, completion of knowledge assessments, and number of questions submitted to healthcare providers.

Adverse events including new infections, hospitalizations, vascular interventions, and amputations were systematically recorded for all participants. All data were collected on standardized case report forms and subsequently entered into a secure electronic database with double-entry verification to ensure data integrity.

Patient allocation and potential bias: Between June 2021 and December 2022, all eligible patients with diabetic foot ulcers were offered WeChat-based remote care. Patient allocation was based on their voluntary consent and demonstrated technical capability to use the WeChat platform. Among 156 eligible patients, those who expressed willingness to use WeChat and demonstrated basic smartphone proficiency during a 10-min orientation session were enrolled in the intervention group (*n* = 73), while those who declined participation or lacked technical capability received standard care and formed the control group (*n* = 58). We acknowledge this non-randomized allocation introduces potential selection bias, which we have addressed through comprehensive statistical adjustments for demographic, clinical, and socioeconomic confounders including age, education level, smartphone ownership, and baseline ulcer severity.

#### Statistical analysis

Continuous variables were presented as mean ± standard deviation for normally distributed data or median (interquartile range) for skewed distributions. Categorical variables were expressed as absolute numbers with percentages. The normality of continuous variables was assessed using the Shapiro-Wilk test. Between-group comparisons were conducted using chi-square test or Fisher’s exact test for categorical variables, independent Student’s t-test for normally distributed continuous variables, and Mann-Whitney U test for non-normally distributed continuous variables.

We employed a three-stage analytical strategy to evaluate the effectiveness of the WeChat-based remote care intervention. First, we performed Kaplan-Meier survival analysis to evaluate time to ulcer healing between intervention groups. The primary outcome was time to complete ulcer healing, with patients censored at their last follow-up visit if healing had not occurred. We used Cox proportional hazards models to examine the association between WeChat-based care intervention and ulcer healing time. The proportional hazards assumption was verified using Schoenfeld residuals (*P* > 0.05 for all variables). Hazard ratios (HRs) with 95% confidence intervals (CIs) were calculated. The multivariate model was adjusted for demographic characteristics (age, sex, education level), clinical parameters (body-mass index, smoking status, glycated haemoglobin, blood pressure), treatment characteristics (economic condition, interventional procedure), and disease severity indicators (Wagner grade, initial wound area). Log-rank tests were used to compare survival curves between groups. Subgroup analyses were conducted according to Wagner grades and interventional procedure status.

Secondly, a repeated measures analysis of variance (ANOVA) was conducted to assess longitudinal changes in quality of life scores (WHOQOL-BREF) and treatment satisfaction (DTSQS) between groups at 3, 6, 12, and 24 months follow-up. Mauchly’s test was employed to assess the sphericity of the data, with Greenhouse-Geisser corrections applied when deemed necessary. *Post-hoc* comparisons were performed using the Bonferroni correction for multiple testing.

Thirdly, we performed stratified analyses to explore the potential for effect modification by interventional procedure status and Wagner grade. For continuous stratification variables, categorical variables were created based on clinically relevant cut-points or tertiles when established thresholds were unavailable. Interaction effects were assessed using likelihood ratio tests. To ensure robust conclusions, we conducted sensitivity analyses by treating the intervention as a categorical variable and calculating *P*-values for trend, allowing us to validate the primary analysis results and examine potential non-linear relationships.

Missing data were handled using multiple imputation with chained equations (MICE) when data were missing at random. We generated 20 imputed datasets and pooled the results using Rubin’s rules. All statistical analyses were performed using R software version 4.1.0 (R Core Team, 2021). Two-sided *P* values less than 0.05 were considered statistically significant. No adjustment was made for multiple comparisons in the stratified analyses, and results should be interpreted as exploratory. This study followed the STROBE guidelines for observational studies.

## Results

Baseline demographic and clinical characteristics were comparable between the WeChat-based intervention and control groups, except for educational level distribution (*P* < 0.001). The intervention group showed a more diverse educational background, including middle school (28.8%) and high school (11.9%) graduates, while the control group consisted only of patients with primary education (51.4%) or no formal education (48.6%). There was a marginally significant difference in economic status (*P* = 0.084), with a higher proportion of patients reporting good economic conditions in the intervention group (81.4% *vs* 68.1%) (Table 1).

**Table 1:** Baseline characteristics and follow-up outcomes of patients by WeChat-based intervention status.

Characteristics	Control group (*n* = 72)	Intervention group (*n* = 59)	*P* value
Demographic and clinical parameters			
Age, years	72.2 (5.2)	72.0 (5.0)	0.823
Sex			0.851
Female	28 (38.9%)	22 (37.3%)	
Male	44 (61.1%)	37 (62.7%)	
BMI, kg/m^2^	20.8 (3.8)	21.2 (3.6)	0.591
HbA1c, %	8.2 (0.6)	8.2 (0.6)	0.876
Blood pressure, mmHg			
Systolic	145.8 (18.5)	146.0 (19.2)	0.938
Diastolic	87.9 (9.6)	88.0 (10.0)	0.96
Wound square, cm^2^	8.4 (2.6)	8.5 (2.7)	0.879
Socioeconomic factors			
Educational level			<0.001
No formal education	35 (48.6%)	12 (20.3%)	
Primary school	37 (51.4%)	22 (37.3%)	
Middle school	0	17 (28.8%)	
High school	0	7 (11.9%)	
University or above	0	1 (1.7%)	
Economic condition			0.084
Poor	23 (31.9%)	11 (18.6%)	
Good	49 (68.1%)	48 (81.4%)	
Smoking status			0.994
Non-smoker	33 (45.8%)	27 (45.8%)	
Smoker	39 (54.2%)	32 (54.2%)	
Clinical characteristics			
Wagner grade			0.922
Grade 1	25 (34.7%)	21 (35.6%)	
Grade 2	26 (36.1%)	20 (33.9%)	
Grade 3	18 (25.0%)	14 (23.7%)	
Grade 4	3 (4.2%)	4 (6.8%)	
Interventional procedure			0.208
No	27 (37.5%)	16 (27.1%)	
Yes	45 (62.5%)	43 (72.9%)	
Quality of life and treatment satisfaction			
WHOQOL-BREF scores			
Baseline	53.6 (8.1)	54.2 (7.4)	0.646
3 months	60.4 (8.4)	63.4 (8.2)	0.041
6 months	61.9 (9.3)	68.3 (8.1)	<0.001
12 months	62.0 (9.7)	69.6 (8.8)	<0.001
24 months	62.4 (10.3)	70.3 (9.5)	<0.001
DTSQs scores			
Baseline	14.1 (2.7)	14.7 (3.5)	0.255
3 months	16.1 (1.5)	20.8 (2.5)	<0.001
6 months	17.3 (2.1)	22.7 (2.9)	<0.001
12 months	18.3 (2.1)	25.0 (3.2)	<0.001
24 months	19.3 (2.2)	26.0 (3.2)	<0.001
Clinical outcome			
Wound healing			0.001
Not healed	26 (36.1%)	7 (11.9%)	
Healed	46 (63.9%)	52 (88.1%)	

**Note:**

Data are mean (SD) or *n* (%). *P* values were calculated using Student’s t-test for continuous variables and $\chi^2$ test or Fisher’s exact test for categorical variables. BMI, body mass index; HbA1c, glycated haemoglobin; WHOQOL-BREF, World Health Organization Quality of Life-BREF questionnaire; DTSQs, Diabetes Treatment Satisfaction Questionnaire status version.

The wound healing rate was significantly higher in the WeChat remote care guidance group compared to the control group (88.1% *vs* 63.9%, *P* = 0.001). Quality of life, as measured by WHOQOL-BREF scores, showed no significant difference at baseline (54.2 ± 7.4 *vs* 53.6 ± 8.1, *P* = 0.646) but demonstrated progressively increasing differences favoring the intervention group throughout the follow-up period. The differences became significant at 3 months (63.4 ± 8.2 *vs* 60.4 ± 8.4, *P* = 0.041) and continued to widen at 6 months (68.3 ± 8.1 *vs* 61.9 ± 9.3, *P* < 0.001), 12 months (69.6 ± 8.8 *vs* 62.0 ± 9.7, *P* < 0.001), and 24 months (70.3 ± 9.5 *vs* 62.4 ± 10.3, *P* < 0.001) (Table 1).

Similarly, treatment satisfaction measured by DTSQs showed comparable baseline scores (14.7 ± 3.5 *vs* 14.1 ± 2.7, *P* = 0.255) but demonstrated significant improvements in the intervention group throughout the follow-up period. The intervention group showed consistently higher satisfaction scores at 3 months (20.8 ± 2.5 *vs* 16.1 ± 1.5, *P* < 0.001), 6 months (22.7 ± 2.9 *vs* 17.3 ± 2.1, P < 0.001), 12 months (25.0 ± 3.2 *vs* 18.3 ± 2.1, *P* < 0.001), and 24 months (26.0 ± 3.2 *vs* 19.3 ± 2.2, *P* < 0.001) (Table 1).

Other baseline characteristics, including age, BMI, HbA1c, blood pressure, wound size, gender distribution, smoking status, and Wagner grade distribution showed no significant differences between the groups (all *P* > 0.05) (Table 1).

Platform analytics revealed consistent engagement among intervention group patients. Educational content was accessed an average of 4.2 ± 1.7 times weekly, with wound care techniques (94.6%), glucose monitoring guidance (87.3%), and infection recognition (78.9%) being the most frequently viewed topics. Patients submitted wound photographs 2.3 ± 1.1 times weekly, with healthcare professionals responding within 15.2 ± 8.7 min on average.

The platform facilitated 127 remote treatment modifications throughout the study period. Medication reminder acknowledgment rate reached 89.4%, while blood glucose monitoring alerts achieved 76.2% compliance within one hour. Weekly group discussions maintained 67.8% participation rate, addressing common questions about infection recognition, dressing techniques, and offloading strategies.

Platform engagement was highest during the first 4 weeks (5.8 daily interactions per patient), stabilizing at 3.2 daily interactions by week 8. Usage patterns were comparable across age groups, with older patients (>65 years) showing slightly lower but substantial engagement (3.7 ± 1.8 *vs* 4.5 ± 1.6 daily interactions, *P* = 0.38).

**Figure 1:**
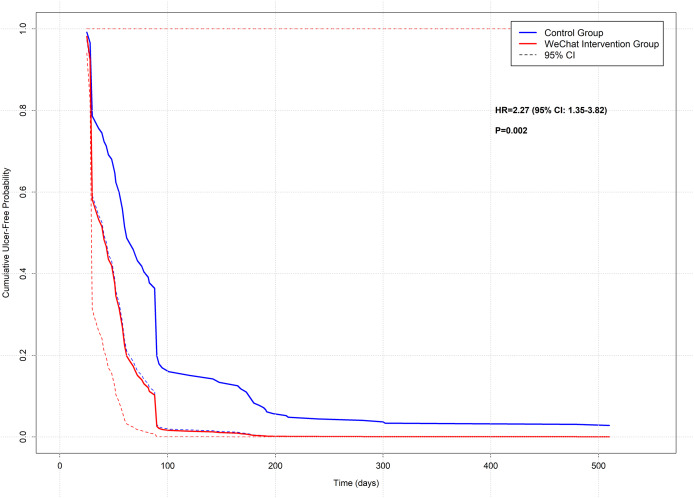
Kaplan-Meier curves comparing diabetic foot ulcer healing between WeChat intervention and control groups. Kaplan-Meier survival analysis demonstrating cumulative ulcer-free probability over time (days) between the WeChat-based remote care intervention group (red line) and the standard care control group (blue line). Dashed lines represent 95% confidence intervals. The WeChat intervention group showed significantly improved healing rates (HR = 2.27, 95% CI [1.35–3.82], *P* = 0.002). The table below the graph indicates the number of patients at risk at specified time points (0, 30, 60, 90, 120, 180, 300, and 510 days). Vertical tick marks would represent censored observations. This analysis was based on a 24-month retrospective cohort study of diabetic foot ulcer patients (*n* = 131).

Kaplan-Meier survival analysis with Cox proportional hazards modeling revealed significant differences in wound healing time between intervention groups. Patients receiving WeChat-based intervention demonstrated significantly faster healing rates compared to the control group (HR = 2.27, 95% CI [1.35–3.82], *P* = 0.0021), with the intervention group showing a more than two-fold increase in healing velocity over the study period (Table 2) (Fig. 1).

**Table 2:** Kaplan-Meier survival analysis of factors associated with time to diabetic foot ulcer healing.

Prognostic factor	Hazard ratio (95% CI)	*P* value
Primary intervention		
WeChat-based care^†^	2.27 [1.35–3.82]	0.002
Demographic characteristics		
Age (per year)	1.00 [0.96–1.05]	0.826
Male sex	0.56 [0.23–1.36]	0.203
Educational level^‡^		
Primary school	0.54 [0.32–0.92]	0.023
Middle school	0.92 [0.45–1.85]	0.806
High school	0.18 [0.05–0.60]	0.005
College or above	1.48 [0.14–15.67]	0.744
Clinical parameters		
BMI (per kg/m^2^)	1.06 [0.99–1.13]	0.081
Current smoking	1.57 [0.71–3.44]	0.263
HbA1c (per %)	0.91 [0.63–1.32]	0.616
Systolic BP (per 10 mmHg)	0.90 [0.72–1.12]	0.348
Diastolic BP (per 10 mmHg)	1.13 [0.77–1.66]	0.525
Treatment and disease characteristics		
Economic status^§^	1.23 [0.72–2.08]	0.451
Interventional procedure^¶^	2.27 [1.27–4.06]	0.006
Wagner grade^‖^		
Grade 2	0.21 [0.07–0.60]	0.004
Grade 3	0.06 [0.01–0.35]	0.002
Grade 4	0.02 [0.002–0.28]	0.003
Wound area (per cm^2^)	1.46 [0.81–2.62]	0.206

**Notes:**

Data are hazard ratios with 95% confidence intervals from multivariate Cox proportional hazards regression analysis. BMI, Body mass index; BP, blood pressure; HbA1c, glycated haemoglobin; CI, confidence interval.

† Reference group is standard care without WeChat intervention.

‡ Reference group is no formal education.

§ Economic status was categorised based on monthly household income.

¶ Reference group is patients without interventional procedures.

‖ Reference group is Wagner Grade 1.

The survival analysis identified several other significant predictors of wound healing time. Interventional procedures were associated with significantly accelerated healing (HR = 2.27, 95% CI [1.27–4.06], *P* = 0.0058), demonstrating a similar magnitude of effect as the WeChat intervention. Wagner grade showed a strong inverse relationship with healing rate, with higher grades associated with progressively longer healing times. Compared to Wagner grade 1, the hazard ratios were 0.21 (95% CI [0.07–0.60], *P* = 0.0037) for grade 2, 0.06 (95% CI [0.01–0.35], *P* = 0.0017) for grade 3, and 0.02 (95% CI [0.002–0.28], *P* = 0.0026) for grade 4, indicating substantially delayed healing with increasing ulcer severity (Table 2).

Educational level emerged as an unexpected predictor of healing time, with both primary education (HR = 0.54, 95% CI [0.32–0.92], *P* = 0.0228) and high school education (HR = 0.18, 95% CI [0.05–0.60], *P* = 0.0052) associated with longer healing times compared to no formal education. Other demographic and clinical characteristics, including age, sex, BMI, smoking status, HbA1c levels, blood pressure, economic condition, and wound size, did not significantly influence healing time in the survival analysis (all *P* > 0.05) (Table 2).

These findings suggest that while WeChat-based intervention and interventional procedures significantly accelerate wound healing, the baseline Wagner grade remains a crucial determinant of healing time. The unexpected association between educational level and healing time warrants further investigation into potential underlying socioeconomic or behavioral factors (Table 2).

Analysis of quality of life outcomes revealed progressive improvements in both WHOQOL-BREF and DTSQS scores over the 24-month follow-up period. At baseline, there were no significant differences between the WeChat remote care guidance and control groups in either WHOQOL-BREF scores (54.19 ± 7.45 *vs* 53.56 ± 8.09, *P* = 0.646) or DTSQS scores (14.73 ± 3.52 *vs* 14.11 ± 2.66, *P* = 0.255) (Table 3).

**Table 3:** Changes in quality of life and treatment satisfaction scores over 24-month follow-up.

Outcome measure and time point	WeChat intervention (*n* = 59)	Control (*n* = 72)	Mean difference (95% CI)	*P* value
WHOQOL-BREF score				
Baseline	54.19 ± 7.45	53.56 ± 8.09	0.63 [−2.08 to 3.34]	0.646
3 months	63.41 ± 8.18	60.40 ± 8.35	3.00 [0.13–5.88]	0.041
6 months	68.32 ± 8.09	61.88 ± 9.28	6.45 [3.40–9.49]	<0.001
12 months	69.64 ± 8.84	61.97 ± 9.68	7.67 [4.44–10.91]	<0.001
24 months	70.31 ± 9.53	62.43 ± 10.32	7.87 [4.41–11.34]	<0.001
DTSQS score				
Baseline	14.73 ± 3.52	14.11 ± 2.66	0.62 [−0.45 to 1.69]	0.255
3 months	20.76 ± 2.53	16.07 ± 1.47	4.69 [3.99–5.39]	<0.001
6 months	22.66 ± 2.87	17.29 ± 2.09	5.37 [4.51–6.23]	<0.001
12 months	24.97 ± 3.21	18.29 ± 2.09	6.67 [5.75–7.60]	<0.001
24 months	25.97 ± 3.21	19.26 ± 2.16	6.70 [5.77–7.64]	<0.001

**Note:**

Data are mean ± SD unless otherwise specified. WHOQOL-BREF, World Health Organization Quality of Life-BREF questionnaire; DTSQS, Diabetes Treatment Satisfaction Questionnaire Status; CI, confidence interval.

**Figure 2:**
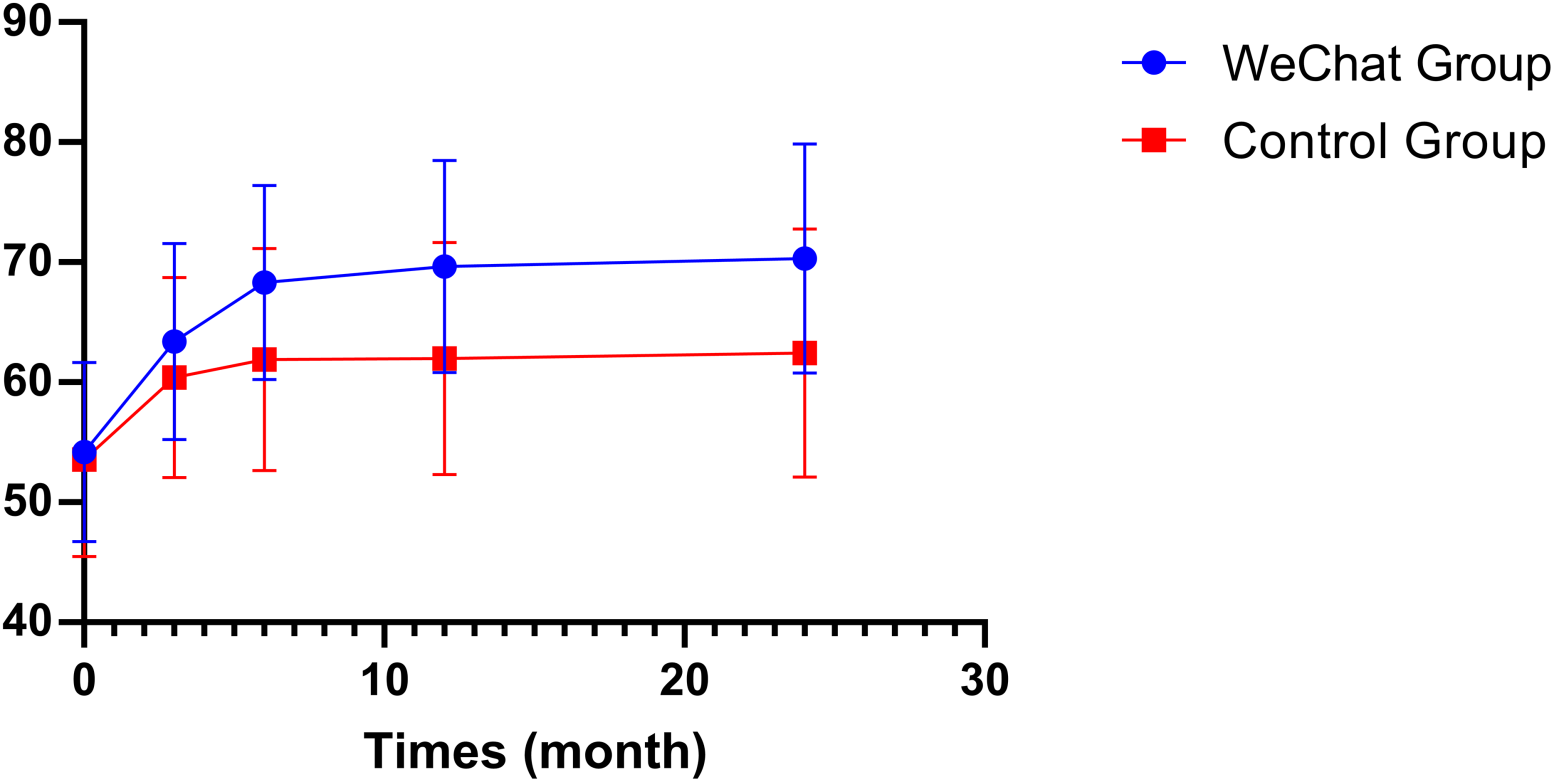
Changes in quality of life scores (WHOQOL-BREF) over the 24-month follow-up period. The graph shows the progression of World Health Organization Quality of Life Brief Version (WHOQOL-BREF) scores at baseline (0 months) and follow-up time points (3, 12, and 24 months) for both the WeChat intervention group (blue circles) and the standard care control group (red squares). Error bars represent standard deviations. The WeChat group demonstrated greater improvement in quality of life scores from baseline, with sustained higher scores throughout the follow-up period. Starting from similar baseline values (approximately 54 points), the WeChat group achieved mean scores of approximately 70 points by 24 months, while the control group stabilized around 62 points.

The WHOQOL-BREF scores showed a significant divergence between groups starting from the 3-month follow-up (63.41 ± 8.18 *vs* 60.40 ± 8.35, mean difference = 3.00, *P* = 0.041). This difference progressively increased at 6 months (68.32 ± 8.09 *vs* 61.88 ± 9.28, mean difference = 6.45, *P* < 0.001), 12 months (69.64 ± 8.84 *vs* 61.97 ± 9.68, mean difference = 7.67, *P* < 0.001), and remained stable through 24 months (70.31 ± 9.53 *vs* 62.43 ± 10.32, mean difference = 7.87, *P* < 0.001) (Table 3) (Fig. 2).

**Figure 3:**
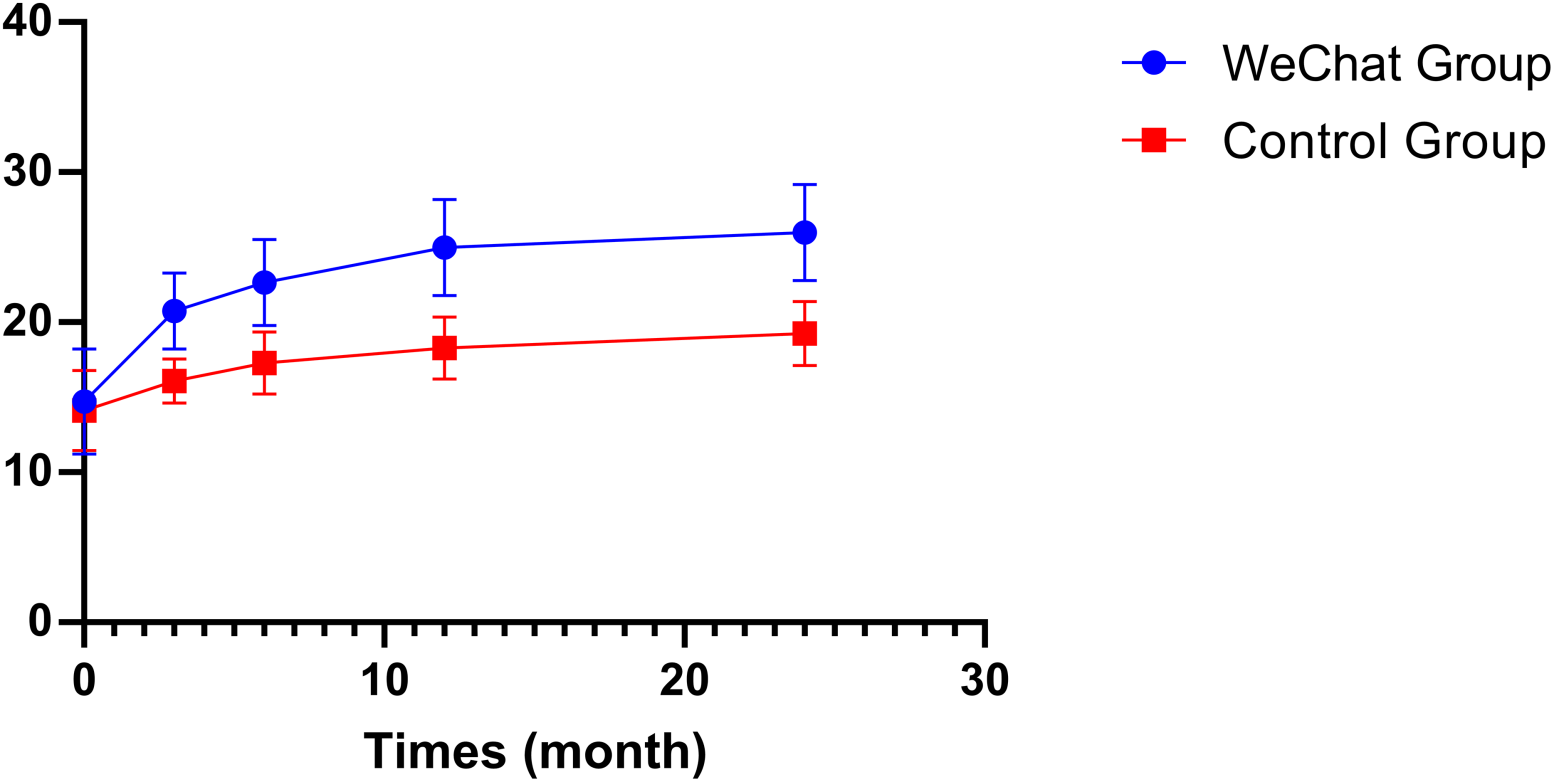
Comparison of diabetic treatment satisfaction (DTSQS) scores between study groups. Diabetic Treatment Satisfaction Questionnaire Status (DTSQS) scores are plotted for the WeChat intervention group (blue circles) and the standard care control group (red squares) at baseline (0 months) and follow-up time points (3, 12, and 24 months). Error bars represent standard deviations. Both groups showed improvement in treatment satisfaction, but the WeChat intervention group demonstrated significantly greater increases, starting from similar baseline values (approximately 14 points) and reaching approximately 26 points by 24 months, compared to the control group’s approximately 19 points. The consistent separation between curves indicates a sustained positive effect of the WeChat-based intervention on patient treatment satisfaction.

Treatment satisfaction, as measured by DTSQS scores, demonstrated even more pronounced differences between groups. Starting from 3 months, the intervention group showed significantly higher satisfaction scores (20.76 ± 2.53 *vs* 16.07 ± 1.47, mean difference = 4.69, *P* < 0.001), with the gap widening at 6 months (22.66 ± 2.87 *vs* 17.29 ± 2.09, mean difference = 5.37, *P* < 0.001), 12 months (24.97 ± 3.21 *vs* 18.29 ± 2.09, mean difference = 6.67, *P* < 0.001), and maintaining through 24 months (25.97 ± 3.21 *vs* 19.26 ± 2.16, mean difference = 6.70, *P* < 0.001) (Table 3) (Fig. 3).

These results demonstrate that the WeChat-based intervention not only significantly improved patients’ quality of life but also substantially enhanced their treatment satisfaction, with both effects becoming more pronounced over time and maintaining stability through the extended follow-up period (Table 4).

**Table 4:** Stratified analysis of WeChat intervention effect on wound healing by treatment modality and ulcer severity.

Stratification factor	*n*	Adjusted HR (95% CI)*	*P* value
Interventional procedure			
Without intervention	43	1.7 [0.6–4.9]	0.359
With intervention	88	3.4 [1.8–6.3]	<0.001
Wagner grade			
Grade 1	46	1.4 [0.5–3.6]	0.503
Grade 2	46	14.3 [5.0–40.7]	<0.001
Grade 3	32	34.2 [4.5–261.3]	<0.001
Grade 4^†^	7	NA	NA

**Notes:**

Data are hazard ratios (HR) with 95% confidence intervals (CI). NA, not available.

* Adjusted for age, sex, education level, body mass index, smoking status, glycated haemoglobin, systolic and diastolic blood pressure, economic condition, and wound square.

† Effect estimate not available due to limited sample size.

Stratified analyses revealed significant effect modifications of the WeChat remote care guidance by both interventional procedure status and Wagner grade. These analyses were adjusted for age, sex, education level, BMI, smoking status, HbA1c, blood pressure, economic condition, and wound square.

In the interventional procedure stratification, the WeChat remote care guidance showed markedly different effects between subgroups. Among patients receiving interventional procedures (*n* = 88), the WeChat remote care guidance significantly accelerated wound healing (HR = 3.4, 95% CI [1.8–6.3], *P* < 0.001), indicating a 240% increase in healing rate. However, in patients without interventional procedures (*n* = 43), the effect was notably attenuated and non-significant (HR = 1.7, 95% CI [0.6–4.9], *P* = 0.359), suggesting a synergistic effect between WeChat remote care guidance and interventional procedures (Table 4).

The impact of the WeChat remote care guidance also varied substantially across Wagner grades, demonstrating a non-linear pattern of effectiveness. For Wagner grade 1 ulcers (*n* = 46), the intervention showed minimal effect (HR = 1.4, 95% CI [0.5–3.6], *P* = 0.503). However, the effectiveness increased dramatically for grade 2 ulcers (*n* = 46), with a 1,330% improvement in healing rate (HR = 14.3, 95% CI [5.0–40.7], *P* < 0.001). The strongest effect was observed in grade 3 ulcers (*n* = 32), showing a substantial increase in healing rate (HR = 34.2, 95% CI [4.5–261.3], *P* < 0.001), although the wide confidence interval suggests some uncertainty in the precise magnitude of this effect. The impact on grade 4 ulcers could not be estimated due to limited sample size (*n* = 7) (Table 4).

These findings suggest that the WeChat remote care guidance may be most beneficial when combined with interventional procedures and particularly effective for moderate to severe ulcers (Wagner grades 2–3), while showing limited benefit for mild ulcers. The extremely large effect sizes in higher Wagner grades, while statistically significant, should be interpreted with caution given the wide confidence intervals (Table 4).

## Discussion

This study appraised the efficacy of WeChat-based remote guidance in expediting the healing of diabetic foot ulcers through a 24-month longitudinal observation. The study encompassed 131 patients afflicted with diabetic foot ulcers, employing a standardised remote management process and a wound assessment methodology. A comprehensive evaluation was conducted utilising the WHOQOL-BREF and DTSQS scales. The study found that the ulcer healing rate of patients receiving WeChat remote guidance was significantly higher (88.1% *vs* 63.9%, *P* = 0.001). Kaplan-Meier survival analysis revealed that WeChat-based intervention was associated with a significantly higher probability of ulcer healing (HR = 2.27, 95% CI [1.35–3.82], *P* = 0.002). This beneficial effect was particularly evident in patients who underwent interventional procedures, where a similar magnitude of improvement was observed (HR = 2.27, 95% CI [1.27–4.06], *P* = 0.006). Notably, the Wagner grade emerged as a crucial prognostic factor, with higher grades associated with progressively decreased healing rates compared to Grade 1 ulcers (Grade 2: HR = 0.21, 95% CI [0.07–0.60], *P* = 0.004; Grade 3: HR = 0.06, 95% CI [0.01–0.35], *P* = 0.002; Grade 4: HR = 0.02, 95% CI [0.002–0.28], *P* = 0.003). Furthermore, the patients’ quality of life and treatment satisfaction exhibited a trend of continuous improvement, with an increase of 7.87 points (*P* < 0.001) and 6.70 points (*P* < 0.001), respectively, in comparison to the control group at the 24-month follow-up.

This study found that WeChat remote guidance significantly improved the healing rate of diabetic foot ulcers (88.1% *vs* 63.9%, *P* = 0.001). This improvement can be attributed to multiple factors. Primarily, the real-time image transmission and online consultation functions of the WeChat platform enabled the medical team to detect and address potential complications in a timely manner, which was reflected in a significant improvement in patient treatment satisfaction (DTSQS score increased from 14.73 ± 3.52 at baseline to 25.97 ± 3.21 at the 24-month follow-up). Factors associated with poor DFU healing include poor diabetes control (Shah et al., 2021). Research has demonstrated that early and intensive glycemic control is instrumental in the healing of diabetic foot ulcers. Dutta, Bhansali & Rastogi (2023) found that patients who achieved rapid glycemic control (HbA1c < 8.15%) within 4 weeks were more likely to achieve ulcer healing within 3 months. This finding aligns with the observations made in the present study, wherein the HbA1c levels at the four-week mark were found to be considerably lower in the intervention group compared to the control group (7.7% *vs* 8.9%, *P* = 0.001).

The effectiveness of WeChat-based interventions has been consistently demonstrated across various healthcare settings. In 2022, a randomized controlled trial utilizing the WeChat platform for telemedicine education and care guidance for parents of children with type 1 diabetes mellitus showed significantly higher WHOQOL-BREF scores in the intervention group compared to the control group six months after discharge (Wang et al., 2024). Similarly, a study on primiparous women undergoing cesarean delivery found that continuity of care through the WeChat platform resulted in significantly higher quality of life scores (SF-36) and a lower rate of complications in the observation group compared to the control group (Shao et al., 2020). Another study involving adults with type 2 diabetes demonstrated that WeChat-based online intervention significantly improved medication adherence in the intervention group (Zhang et al., 2024).

These findings align with our study’s results, where the team of specially trained vascular surgeons and vascular care specialists provided expert guidance through a standardised remote management process, crucial for ensuring treatment adherence. In particular, among patients who underwent interventional treatment (*n* = 88), the WeChat remote care guidance significantly accelerated wound healing (HR: 2.27, 95% CI [1.35–3.82], *P* < 0.001), indicating that remote guidance improves outcomes by increasing patient compliance with postoperative treatment regimens. The systematic educational modules and Q&A service covered key content such as guidance on drug use, blood glucose monitoring, wound care and infection prevention. This comprehensive educational support enhanced patients’ self-management capabilities, as reflected in the significant improvement in the quality of life score (WHOQOL-BREF score increased from 54.19 ± 7.45 at baseline to 70.31 ± 9.53 at the 24-month follow-up). Furthermore, the impact of the WeChat remote care guidance was particularly pronounced among patients with Wagner grade 2–3 ulcers (HR = 14.3–34.2, *P* < 0.001). This observation may be attributed to the necessity for enhanced monitoring and standardised treatment of these patients, along with their higher compliance with drug therapy. In addition, Lubis, Marshal & Ali Syahputra (2022) also confirmed that good glycemic control is an important predictor of diabetic foot ulcer healing, and that for every 1% decrease in HbA1c, the likelihood of ulcer healing significantly increases.

Diabetic foot ulcers are strongly associated with an increased risk of death (Walsh et al., 2016). According to the study, among patients with diabetes, 5.0% of those with new diabetic foot ulcers died within 12 months, and 42.2% died within 5 years. Although in this study we excluded patients with serious illnesses and those who died during follow-up, some of the patients encountered during the implementation of our WeChat REMOTE CARE GUIDELINES needed timely medical care and arrived at the health facility on time, as advised by our team.

The clinical value of this study is reflected in three aspects. Firstly, the study systematically evaluated the differential impact of WeChat remote guidance on patients with diabetic foot of different Wagner grades through long-term follow-up of 24 months and stratified analysis. This is the first study to do so, and the findings provide a more precise intervention strategy for clinical practice. The study found that WeChat remote care guidance was most effective in patients with Wagner grade 2–3, suggesting that this finding can help optimise the allocation of medical resources and develop personalised remote management plans for patients of different severities. Secondly, the study confirmed the synergistic effect between WeChat remote guidance and interventional therapy, providing a new idea for a comprehensive treatment model for diabetic foot. In light of these findings, it is recommended that WeChat remote guidance be employed as a supplement to standard treatment in clinical practice, particularly for patients with moderate to severe ulcers undergoing interventional therapy. Moreover, the standardised remote management process and evaluation methods utilised in the study can serve as a template for other medical institutions seeking to develop telemedicine services. Future research directions could include the exploration of an artificial intelligence-assisted wound assessment system and the optimisation of remote management strategies for patients from diverse socioeconomic backgrounds.

The efficacy of offloading procedures is paramount for the healing of DFU (Armstrong, Boulton & Bus, 2017). However, patient adherence to prescribed offloading interventions remains a substantial challenge. Research has demonstrated that patients wear their prescribed footwear or offloading devices for only a proportion of their total ambulatory time, considerably affecting treatment outcomes (Bus et al., 2016; Bus, 2016). This poor adherence has been extensively documented, with compelling evidence indicating that non-adherent patients experience significantly worse healing outcomes. The effectiveness of offloading interventions is invariably confounded by levels of adherence, with even the most effective device failing if not worn consistently. This challenge is particularly evident in the case of removable devices, where studies have reported limited wearing time during daily activities (Bus et al., 2016; Bus, 2016). Non-removable devices can overcome this adherence issue, though they present their own set of challenges. To address this critical issue, healthcare providers must implement regular monitoring and provide timely professional guidance on proper device usage. Strategies such as the provision of more attractive or specific offloading footwear for indoor use and the enhancement of patients’ perception of footwear benefits may assist in the enhancement of adherence to prescribed interventions. While our WeChat-based remote care intervention demonstrated significant benefits for diabetic foot ulcer management, we recognize opportunities for enhancement in future iterations. Specifically, our platform could be strengthened by incorporating more comprehensive offloading therapy components. Offloading is recognized as a cornerstone of effective diabetic foot ulcer management, with proper pressure redistribution being essential for healing promotion. Future versions of our WeChat platform should include detailed offloading education modules with video demonstrations of proper device application, personalized reminders for device usage, and remote compliance monitoring capabilities. Healthcare providers could remotely assess proper offloading technique through image sharing, and the platform could facilitate regular documentation of wearing time. This enhancement would address a critical aspect of diabetic foot care, as non-compliance with offloading remains a significant barrier to ulcer healing. By integrating structured offloading education and monitoring into our remote care platform, we could potentially further improve healing outcomes beyond what was observed in the current study.

The present study boasts several notable strengths. Firstly, the study design incorporates a 24-month long-term follow-up period, which significantly surpasses the observation period of previous similar studies and provides a robust foundation for evaluating the sustained effect of WeChat remote guidance. Secondly, the establishment of a multidisciplinary team comprising vascular surgeons and professional vascular care experts, in addition to the implementation of a standardised remote management process and wound assessment method, ensures the quality and consistency of the intervention. The data analysis strategy comprised a three-stage approach. The intervention effect was evaluated using a Cox proportional hazards model, repeated measures analysis of variance was used to track longitudinal changes in quality of life and treatment satisfaction, and stratified analysis was performed to explore the intervention effect in different subgroups. In particular, when dealing with confounding factors, we comprehensively adjusted variables including age, gender, education level, BMI, smoking status, blood glucose control, blood pressure, economic status and initial wound area, significantly improving the reliability of the research results. In addition, we used internationally recognised assessment tools (WHOQOL-BREF and DTSQs scale), and processed missing data using multiple imputation methods, which further enhanced the scientific nature and rigor of the study.

The present study is subject to several limitations. Firstly, the inclusion criteria necessitated proficiency in the utilisation of WeChat among patients and primary caregivers, which may restrict the generalisability of the findings to patient populations with limited digital proficiency or restricted access to smart devices. Secondly, the study excluded critically ill patients requiring immediate hospitalisation, patients with severe cardiac, hepatic or renal dysfunction, and patients with mental disorders. Consequently, the findings of this study may not be applicable to these special populations. As a single-centre retrospective cohort study, the generalisability of its results is limited and further validation is needed through multi-centre prospective studies. In addition, as the study subjects were all from Fujian Province in China, caution is needed when generalising the findings to other ethnicities or regions. As a limitation inherent to observational studies, this study can only confirm the association between WeChat remote guidance and diabetic foot ulcer healing, but cannot determine a causal relationship. The non-randomized patient allocation based on voluntary participation may have introduced selection bias, as patients choosing WeChat-based care might have been more motivated or technologically proficient, potentially influencing outcomes independent of the intervention. Finally, although several measurable confounders were adjusted for in the analysis, the potential for unmeasured confounders, such as patient compliance and family support, which may affect the interpretation of the results, remains.

## Conclusion

This study demonstrates that WeChat remote guidance is associated with the healing rate of diabetic foot ulcers, particularly in patients with Wagner 2–3 ulcers and those undergoing interventional treatment. This intervention may provide a viable solution for the management of diabetic foot ulcers in areas with limited medical resources. The findings of the present study lend support to the notion that WeChat remote guidance can serve as an effective adjunct to standard treatment, particularly in cases where long-term follow-up and continuous management are required. In the future, the value of promoting this intervention model and exploring personalised remote management strategies for different patient groups could be further substantiated by large-scale multicentre randomised controlled trials.

## Supplemental Information

10.7717/peerj.20624/supp-1 Supplemental Information 1 raw data.

10.7717/peerj.20624/supp-2 Supplemental Information 2 STROBE checklist.

10.7717/peerj.20624/supp-3 Supplemental Information 3 Data annotation.

## Additional Information and Declarations

### Data Availability

The following information was supplied regarding data availability:

The raw data is available in the Supplemental Files.

## References

[ref-1] Armstrong DG, Boulton AJM, Bus SA (2017). Diabetic foot ulcers and their recurrence. New England Journal of Medicine.

[ref-2] Bus SA (2016). The role of pressure offloading on diabetic foot ulcer healing and prevention of recurrence. Plastic & Reconstructive Surgery.

[ref-3] Bus SA, van Deursen RW, Armstrong DG, Lewis JE, Caravaggi CF, Cavanagh PR, International Working Group on the Diabetic Foot (2016). Footwear and offloading interventions to prevent and heal foot ulcers and reduce plantar pressure in patients with diabetes: a systematic review. Diabetes/Metabolism Research and Reviews.

[ref-4] Chen X, Zhou X, Li H, Li J, Jiang H (2020). The value of WeChat application in chronic diseases management in China. Computer Methods and Programs in Biomedicine.

[ref-5] Dutta A, Bhansali A, Rastogi A (2023). Early and intensive glycemic control for diabetic foot ulcer healing: a prospective observational nested cohort study. The International Journal of Lower Extremity Wounds.

[ref-6] Ghanassia E, Villon L, Thuan Dit Dieudonne JF, Boegner C, Avignon A, Sultan A (2008). Long-term outcome and disability of diabetic patients hospitalized for diabetic foot ulcers: a 6.5-year follow-up study. Diabetes Care.

[ref-7] Lubis MIP, Marshal, Ali Syahputra M (2022). Association of glycosyllated hemoglobin (Hba1c) levels to the severity of diabetic foot ulcer in type 2 diabetes mellitus patients in RSUP. H. Adam Malik Medan. Sumatera Medical Journal.

[ref-8] Oliveira-Cortez A, Rodrigues Ferreira I, Luiza Nunes Abreu C, de Oliveira Bosco Y, Kummel Duarte C, Nogueira Cortez D (2023). Incidence of the first diabetic foot ulcer: a systematic review and meta-analysis. Diabetes Research and Clinical Practice.

[ref-18] R Core Team (2021). R: a language and environment for statistical computing.

[ref-9] Saisho Y (2018). Use of diabetes treatment satisfaction questionnaire in diabetes care: importance of patient-reported outcomes. International Journal of Environmental Research and Public Health.

[ref-10] Shah GA, Keerio NH, Khanzada AA, Joyo MR, Ahmed N, Afzal T, Noor SS (2021). Study to determine the complications and treatment of diabetic foot ulcer. Journal of Pharmaceutical Research International.

[ref-11] Shao F, He Z, Zhu Z, Wang X, Zhang J, Shan J, Pan J, Wang H (2020). Internet influence of assisted reproduction technology centers in China: qualitative study based on WeChat official accounts. Journal of Medical Internet Research.

[ref-12] Walsh JW, Hoffstad OJ, Sullivan MO, Margolis DJ (2016). Association of diabetic foot ulcer and death in a population-based cohort from the United Kingdom. Diabetic Medicine.

[ref-13] Wang H, Wu H, Zhang Y, Ding Q, Zhang Y (2024). WeChat continuity nursing on postpartum depression and quality of life in primipara undergoing cesarean delivery. Alternative Therapies, Health and Medicine.

[ref-14] Yang J, Yang H, Wang Z, Wang X, Wang Y, Yu X, Liu L (2021). Self-management among type 2 diabetes patients via the WeChat application: a systematic review and meta-analysis. Journal of Clinical Pharmacy and Therapeutics.

[ref-15] Zaidi A, Elsayed B, Jemmieh K, Nawaz A, Eledrisi M, Chowdhury MEH, Zughaier SM, Hasan A, Alfkey R (2024). Perspective chapter: epidemiology and risk factors of diabetic foot ulcer. Diabetic Foot Ulcers—Pathogenesis, Innovative Treatments and AI Applications.

[ref-16] Zhang B, Kalampakorn S, Powwattana A, Sillabutra J, Liu G (2024). A transtheoretical model-based online intervention to improve medication adherence for Chinese adults newly diagnosed with type 2 diabetes: a mixed-method study. Journal of Primary Care & Community Health.

[ref-17] Zhang P, Lu J, Jing Y, Tang S, Zhu D, Bi Y (2017). Global epidemiology of diabetic foot ulceration: a systematic review and meta-analysis. Annals of Medicine.

