# Supplementary material for: Association between WeChat-based remote care guidance and diabetic foot ulcer healing: a retrospective cohort study
Source: PeerJ. 2026 Jan 9;14:e20624. doi: 10.7717/peerj.20624 (PMC12794630; doi:10.7717/peerj.20624)
Supplement: Supplemental Information 2 [file peerj-14-20624-s002.docx]

STROBE Statement—checklist of items that should be included in reports of observational studies

|  | Item No. | Recommendation | Page  No. | Relevant text from manuscript |
| --- | --- | --- | --- | --- |
| **Title and abstract** | 1 | (*a*) Indicate the study’s design with a commonly used term in the title or the abstract | 1 | Association Between WeChat-Based Remote Care Guidance and Diabetic Foot Ulcer Healing: A Retrospective Cohort Study" |
|  |  | (*b*) Provide in the abstract an informative and balanced summary of what was done and what was found | 1 | "Methods: A retrospective cohort study was conducted at Fujian Maternity and Child Health Hospital between June 2021 and December 2022, with follow-up until December 2024. Among 131 eligible patients with diabetic foot ulcers (Wagner grades 1-4), 59 received WeChat-based guidance (intervention) while 72 received standard care (control). Primary outcomes included wound healing rate and time-to-healing." |
| Introduction | | | |  |
| Background/rationale | 2 | Explain the scientific background and rationale for the investigation being reported | 1 | "Diabetic foot ulcers (DFUs) affect approximately 6.3% of the global diabetic population and significantly impact quality of life and healthcare costs. Among diabetic patients, 19-34% will develop foot ulcers in their lifetime, with a 5-year recurrence rate of 70%. These ulcers frequently lead to lower extremity amputations, with over 50% of non-traumatic amputations attributed to diabetic foot complications." |
| Objectives | 3 | State specific objectives, including any prespecified hypotheses | 1 | This study aimed to evaluate the effectiveness of WeChat-based remote care guidance as a supplement to standard care for patients with diabetic foot ulcers. We hypothesized that patients receiving WeChat-based remote care guidance in addition to standard care would demonstrate better ulcer healing outcomes, improved self-management behaviors, and greater treatment satisfaction compared to those receiving standard care alone." |
| Methods | | | |  |
| Study design | 4 | Present key elements of study design early in the paper | 3 | "Study Design and Setting: We conducted a retrospective cohort study at Fujian Maternity and Child Health Hospital between January 2022 and May 2023. This study examined outcomes in patients with diabetic foot ulcers who received either standard care alone or standard care supplemented with WeChat-based remote monitoring." |
| Setting | 5 | Describe the setting, locations, and relevant dates, including periods of recruitment, exposure, follow-up, and data collection | 3 | "We conducted a retrospective cohort study at Fujian Maternity and Child Health Hospital between January 2022 and May 2023... Patient recruitment was conducted through both outpatient clinics and inpatient departments. All eligible patients with diabetic foot ulcers were offered the option to participate in the WeChat-based remote care program." |
| Participants | 6 | (*a*) *Cohort study*—Give the eligibility criteria, and the sources and methods of selection of participants. Describe methods of follow-up  *Case-control study*—Give the eligibility criteria, and the sources and methods of case ascertainment and control selection. Give the rationale for the choice of cases and controls  *Cross-sectional study*—Give the eligibility criteria, and the sources and methods of selection of participants | 3 | "Inclusion criteria were: (1) age ≥18 years; (2) clinically diagnosed diabetic foot ulcers (Wagner grades 1-4); (3) clinically stable condition not requiring immediate hospitalization; and (4) willingness to provide informed consent. For the intervention group, an additional criterion was proficiency in WeChat use by the patient or a primary caregiver. Exclusion criteria included: (1) Wagner grade 5 ulcers; (2) severe cardiac, hepatic, or renal dysfunction; (3) psychiatric disorders preventing compliance; (4) terminal malignancy; (5) life expectancy shorter than the study period; and (6) inability to attend follow-up visits." |
|  |  | (*b*) *Cohort study*—For matched studies, give matching criteria and number of exposed and unexposed  *Case-control study*—For matched studies, give matching criteria and the number of controls per case | N/A | Not applicable as this was not a matched study design |
| Variables | 7 | Clearly define all outcomes, exposures, predictors, potential confounders, and effect modifiers. Give diagnostic criteria, if applicable | 4 | "The exposure was defined as participation in the WeChat-based remote care program. Primary outcomes were ulcer healing rate at 24 weeks and time-to-healing. Secondary outcomes included changes in Diabetic Foot Self-Care Behavior Scale (DFSBS) scores, diabetes treatment satisfaction (measured by DTSQ), and quality of life (measured by WHOQOL-BREF)." |
| Data sources/ measurement | 8* | For each variable of interest, give sources of data and details of methods of assessment (measurement). Describe comparability of assessment methods if there is more than one group | 4-5 | "Diabetic foot ulcer assessment was performed by trained wound care specialists during scheduled clinic visits. Complete healing was defined as full epithelialization without drainage maintained for at least two weeks. Ulcer size was measured using digital planimetry with sterile transparent film tracings... Diabetic foot self-care behaviors were evaluated using the validated Diabetic Foot Self-Care Behavior Scale (DFSBS). This 16-item instrument assesses foot inspection, footwear practices, foot hygiene, and treatment adherence." |
| Bias | 9 | Describe any efforts to address potential sources of bias | 6 | \| "We acknowledge that this non-randomized allocation introduces potential selection bias, which we addressed through comprehensive statistical adjustments for demographic, clinical, and socioeconomic confounders including age, education level, smartphone ownership, and baseline ulcer severity. To minimize assessment bias, wound evaluation was performed by specialists who were not directly involved in the intervention delivery." \| \| --- \| |
| Study size | 10 | Explain how the study size was arrived at | 3 | "Based on previous studies in our center showing a standard care healing rate of approximately 60% at 24 weeks, we calculated that a minimum of 58 patients per group would provide 80% power to detect a clinically meaningful 20% increase in healing rate (from 60% to 80%) with a two-sided alpha of 0.05." |

Continued on next page

| Quantitative variables | 11 | Explain how quantitative variables were handled in the analyses. If applicable, describe which groupings were chosen and why | 6 | "Continuous variables were presented as mean ± standard deviation for normally distributed data or median (interquartile range) for skewed distributions. Categorical variables were expressed as numbers with percentages. The normality of continuous variables was assessed using the Shapiro-Wilk test." |
| --- | --- | --- | --- | --- |
| Statistical methods | 12 | (*a*) Describe all statistical methods, including those used to control for confounding | 6-7 | Between-group comparisons were performed using Student's t-test or Mann-Whitney U test for continuous variables and chi-square or Fisher's exact test for categorical variables. Time-to-healing was analyzed using Kaplan-Meier curves and Cox proportional hazards models. The multivariate model was adjusted for demographic characteristics, clinical parameters, treatment characteristics, and disease severity indicators." |
|  |  | (*b*) Describe any methods used to examine subgroups and interactions | 7 | "We performed stratified analyses to explore potential effect modification by interventional procedure status and Wagner grade. For continuous stratification variables, categorical variables were created based on clinically relevant cut-points. Interaction effects were assessed using likelihood ratio tests." |
|  |  | (*c*) Explain how missing data were addressed | 7 | \| "Missing data were handled using multiple imputation with chained equations (MICE) when data were missing at random. We generated 20 imputed datasets and pooled the results using Rubin's rules." \| \| --- \| |
|  |  | (*d*) *Cohort study*—If applicable, explain how loss to follow-up was addressed  *Case-control study*—If applicable, explain how matching of cases and controls was addressed  *Cross-sectional study*—If applicable, describe analytical methods taking account of sampling strategy | 7 | "Five patients in the intervention group and seven in the control group were lost to follow-up. Following the intention-to-treat principle, we included all patients in the analysis, with the last observation carried forward for those lost to follow-up." |
|  |  | (*e*) Describe any sensitivity analyses | 7 | \| "To ensure robust conclusions, we conducted sensitivity analyses using complete case analysis and by treating the intervention as a categorical variable, allowing us to validate the primary analysis results." \| \| --- \| |
| Results | | | | |
| Participants | 13* | (a) Report numbers of individuals at each stage of study—eg numbers potentially eligible, examined for eligibility, confirmed eligible, included in the study, completing follow-up, and analysed | 8 | "Of 156 patients assessed for eligibility, 131 met the inclusion criteria and were included in the analysis. The WeChat-based remote guidance group included 59 patients, while the standard care group included 72 patients." |
|  |  | (b) Give reasons for non-participation at each stage | 8 | "Twenty-five patients were excluded for the following reasons: Wagner grade 5 ulcers (n=8), severe comorbidities (n=7), unable to provide informed consent (n=5), and declined participation (n=5)." |
|  |  | (c) Consider use of a flow diagram | Fig1 | \| "Figure 1: Flow diagram of patient recruitment and follow-up" \| \| --- \| |
| Descriptive data | 14* | (a) Give characteristics of study participants (eg demographic, clinical, social) and information on exposures and potential confounders | 8-9 | \| "Table 1 presents the baseline demographic and clinical characteristics of study participants. The mean age was 62.4 ± 9.7 years in the WeChat group and 65.1 ± 8.3 years in the control group (p=0.10). The groups were generally comparable, though the WeChat group had a higher proportion of participants with secondary education or above (40.7% vs. 15.3%, p<0.001)." \| \| --- \| |
|  |  | (b) Indicate number of participants with missing data for each variable of interest | 9 | "Complete data were available for 54 patients (91.5%) in the WeChat group and 65 patients (90.3%) in the control group. Missing data were primarily in quality of life assessments (n=7) and self-care behavior measurements (n=5)." |
|  |  | (c) *Cohort study*—Summarise follow-up time (eg, average and total amount) | 8 | The median follow-up duration was 20 weeks (IQR 12-24) in the WeChat group and 22 weeks (IQR 14-24) in the control group." |
| Outcome data | 15* | *Cohort study*—Report numbers of outcome events or summary measures over time | *9* | \| "The complete wound healing rate at 24 weeks was significantly higher in the WeChat group compared to the standard care group (81.4% vs. 61.1%, p=0.01). Median time-to-healing was shorter in the WeChat group (10.2 weeks, 95% CI: 8.6-12.4) than in the standard care group (16.8 weeks, 95% CI: 14.3-19.2, p<0.001)." \| \| --- \| |
|  |  | *Case-control study—*Report numbers in each exposure category, or summary measures of exposure | *N/A* | Not applicable as this was not a matched study design |
|  |  | *Cross-sectional study—*Report numbers of outcome events or summary measures | *N/A* | Not applicable as this was not a matched study design |
| Main results | 16 | (*a*) Give unadjusted estimates and, if applicable, confounder-adjusted estimates and their precision (eg, 95% confidence interval). Make clear which confounders were adjusted for and why they were included | 10 | \| "In the unadjusted analysis, the hazard ratio for healing was 2.12 (95% CI: 1.41-3.18, p<0.001) for the WeChat group compared to the standard care group. After adjusting for age, sex, education level, BMI, diabetes duration, HbA1c, Wagner grade, and initial wound area, the association remained significant with an adjusted hazard ratio of 1.89 (95% CI: 1.23-2.91, p=0.004)." \| \| --- \| |
|  |  | (*b*) Report category boundaries when continuous variables were categorized | 10 | "For stratified analyses, Wagner grades were categorized as mild (grades 1-2) or moderate-to-severe (grades 3-4). Age was categorized as <65 or ≥65 years based on the median age of our cohort." |
|  |  | (*c*) If relevant, consider translating estimates of relative risk into absolute risk for a meaningful time period | 9 | \| "The absolute risk difference in healing rates at 24 weeks was 20.3% (95% CI: 4.9-35.7%), with a number needed to treat of 5 (95% CI: 3-21)." \| \| --- \| |

Continued on next page

| Other analyses | 17 | Report other analyses done—eg analyses of subgroups and interactions, and sensitivity analyses | 11 | "In stratified analyses, the effect of WeChat-based guidance was more pronounced among patients with Wagner grades 3-4 (adjusted HR=2.36, 95% CI: 1.45-3.84, p<0.001) compared to those with Wagner grades 1-2 (adjusted HR=1.42, 95% CI: 0.78-2.59, p=0.25; p-interaction=0.03). Similarly, patients who underwent interventional procedures showed greater benefit from the WeChat intervention (adjusted HR=2.58, 95% CI: 1.56-4.27, p<0.001) compared to those who did not (adjusted HR=1.21, 95% CI: 0.64-2.30, p=0.56; p-interaction=0.02)." |
| --- | --- | --- | --- | --- |
| Discussion | | | | |
| Key results | 18 | Summarise key results with reference to study objectives | 12 | \| "This retrospective cohort study found that WeChat-based remote care guidance was associated with better diabetic foot ulcer healing outcomes compared to standard care alone. Specifically, patients in the WeChat group showed higher healing rates at 24 weeks (81.4% vs. 61.1%) and shorter median healing times (10.2 vs. 16.8 weeks). The intervention appeared particularly beneficial for patients with moderate-to-severe ulcers (Wagner grades 3-4) and those undergoing interventional procedures." \| \| --- \| |
| Limitations | 19 | Discuss limitations of the study, taking into account sources of potential bias or imprecision. Discuss both direction and magnitude of any potential bias | 14 | \| "This study has several limitations. First, as a retrospective cohort study, it is subject to selection bias. Patients who opted to use the WeChat platform might differ from those who did not in ways that could influence outcomes, such as technology literacy, socioeconomic status, or health-seeking behavior. While we adjusted for measured confounders, unmeasured confounding cannot be ruled out. Second, the single-center design limits generalizability. Third, the non-randomized nature of the intervention prevents establishing causality." \| \| --- \| |
| Interpretation | 20 | Give a cautious overall interpretation of results considering objectives, limitations, multiplicity of analyses, results from similar studies, and other relevant evidence | 13 | \| "Our findings suggest that WeChat-based remote care guidance may be a valuable adjunct to standard care for diabetic foot ulcers. The observed associations with improved healing rates and shorter healing times are consistent with previous studies of mobile health interventions in chronic wound care. However, the magnitude of benefit we observed exceeds that reported in some prior studies, which may reflect the intensive nature of our intervention or potential residual confounding... While our findings are promising, they should be interpreted cautiously given the study's observational nature." \| \| --- \| |
| Generalisability | 21 | Discuss the generalisability (external validity) of the study results | 14 | "The generalizability of our findings may be limited to settings with high smartphone penetration and familiarity with social media platforms. Our patient population was predominantly urban Chinese adults with access to specialized diabetes care, and results may differ in rural populations or different healthcare systems. Furthermore, the feasibility of implementing similar interventions depends on local digital literacy, healthcare infrastructure, and provider availability." |
| Other information | |  | | |
| Funding | 22 | Give the source of funding and the role of the funders for the present study and, if applicable, for the original study on which the present article is based | 15 | \| "Funding: This research received no specific grant from any funding agency in the public, commercial, or not-for-profit sectors." \| \| --- \| |

*Give information separately for cases and controls in case-control studies and, if applicable, for exposed and unexposed groups in cohort and cross-sectional studies.

**Note:** An Explanation and Elaboration article discusses each checklist item and gives methodological background and published examples of transparent reporting. The STROBE checklist is best used in conjunction with this article (freely available on the Web sites of PLoS Medicine at http://www.plosmedicine.org/, Annals of Internal Medicine at http://www.annals.org/, and Epidemiology at http://www.epidem.com/). Information on the STROBE Initiative is available at www.strobe-statement.org.
